# Neuromuscular blockade and airway management during endotracheal intubation in Brazilian intensive care units: a national survey

**DOI:** 10.5935/0103-507X.20200073

**Published:** 2020

**Authors:** Pedro Vitale Mendes, Bruno Adler Maccagnan Pinheiro Besen, Fabio Holanda Lacerda, João Gabriel Rosa Ramos, Leandro Utino Taniguchi

**Affiliations:** 1 Emergency Medicine Discipline, Hospital das Clínicas, Faculdade de Medicina, Universidade de São Paulo - São Paulo (SP), Brazil.; 2 Oncological Intensive Care Unit, Hospital São Luiz Rede D’Or - São Paulo (SP), Brazil.; 3 Intensive Care Unit, Hospital da Luz - São Paulo (SP), Brazil.; 4 Intensive Care Unit, Hospital São Rafael - Salvador (BA), Brazil.; 5 Hospital Sírio-Libanês - São Paulo (SP), Brazil.

**Keywords:** Airway management, Intubation, Neuromuscular blockade, Hypnotics and sedatives, Intensive care units, Brazil, Manuseio das vias aéreas, Intubação, Bloqueio neuromuscular, Hipnóticos e sedativos, Unidades de terapia intensiva, Brasil

## Abstract

**Objective:**

To describe the use of neuromuscular blockade as well as other practices among Brazilian physicians in adult intensive care units.

**Methods:**

An online national survey was designed and administered to Brazilian intensivists. Questions were selected using the Delphi method and assessed physicians’ demographic data, intensive care unit characteristics, practices regarding airway management, use of neuromuscular blockade and sedation during endotracheal intubation in the intensive care unit. As a secondary outcome, we applied a multivariate analysis to evaluate factors associated with the use of neuromuscular blockade.

**Results:**

Five hundred sixty-five intensivists from all Brazilian regions responded to the questionnaire. The majority of respondents were male (65%), with a mean age of 38 ( 8.4 years, and 58.5% had a board certification in critical care. Only 40.7% of the intensivists reported the use of neuromuscular blockade during all or in more than 75% of endotracheal intubations. In the multivariate analysis, the number of intubations performed monthly and physician specialization in anesthesiology were directly associated with frequent use of neuromuscular blockade. Etomidate and ketamine were more commonly used in the clinical situation of hypotension and shock, while propofol and midazolam were more commonly prescribed in the situation of clinical stability.

**Conclusion:**

The reported use of neuromuscular blockade was low among intensivists, and sedative drugs were chosen in accordance with patient hemodynamic stability. These results may help the design of future studies regarding airway management in Brazil.

## Abstract (pt)

**Objetivo:**

Descrever o uso do bloqueio neuromuscular e de outras práticas entre os médicos brasileiros atuantes em unidades de terapia intensiva para pacientes adultos.

**Métodos:**

Um levantamento nacional on-line foi aplicado a intensivistas brasileiros. As questões foram selecionadas utilizando o método Delphi e avaliavam os dados demográficos dos médicos, as características da unidade de terapia intensiva, as práticas relativas ao manuseio das vias aéreas e o uso de bloqueio neuromuscular e sedação durante a intubação endotraqueal na unidade de terapia intensiva. Como desfecho secundário, aplicamos uma análise multivariada para avaliar fatores associados com o uso do bloqueio neuromuscular.

**Resultados:**

Responderam ao questionário 565 intensivistas de todas as regiões do país. A maioria dos que responderam era homens (65%), com média de idade de 38 ± 8,4 anos, e 58,5% dos participantes tinham título de especialista em terapia intensiva. Apenas 40,7% dos intensivistas relataram o uso de bloqueio neuromuscular durante todas ou em mais de 75% das intubações endotraqueais. Na análise multivariada, o número de intubações realizadas por mês e a especialização do médico em anestesiologia se associaram diretamente com o uso frequente de bloqueio neuromuscular. Etomidato e cetamina foram utilizados mais comumente na situação clínica de hipotensão e choque, enquanto propofol e midazolam foram mais comumente prescritos em situações de estabilidade hemodinâmica.

**Conclusão:**

O relato de uso de bloqueio neuromuscular foi baixo entre intensivistas, e os fármacos sedativos foram escolhidos segundo a estabilidade hemodinâmica do paciente. Estes resultados podem ajudar no delineamento de futuros estudos relativos ao manuseio das vias aéreas no Brasil.

## INTRODUCTION

Endotracheal intubation (EI) is associated with a high rate of complications in the intensive care unit (ICU). Approximately 10% of all EI fulfill the criteria for a difficult airway, and the incidence of any complication may be as high as 40% in some cohorts of critically ill patients.^(1-3)^ Severe complications are also common: hypoxemia, hypotension and cardiac arrest may occur in 26%, 25% and 2% of cases, respectively.^(4)^ In an attempt to reduce such complications, several international societies have published guidelines with recommendations regarding patient positioning, preoxygenation and use of sedative agents.^(5-7)^ More specifically, the routine use of neuromuscular blockade (NMB) and rapid sequence induction is endorsed as an important adjunctive therapy to facilitate airway visualization and reduce procedure-related complications.^(8)^

In the operating room, several clinical trials and a systematic review have indicated the superiority of the routine use of NMB during airway management.^(9)^ However, in the ICU setting, this recommendation of routine use of NMB during EI is supported only by the evidence from the operating room and observational studies.^(9-11)^ Moreover, bedside practice is highly variable among intensivists, and the use of NMB in EI varies from 20 to 90% in different cohorts.^(12-14)^

Therefore, considering the paucity of data regarding the use of NMB during EI, as well as other airway management practices used in the ICU, we decided to perform a national survey among adult intensive care physicians in Brazil.

The objective of this survey was to describe the use of NMB during orotracheal intubation in the ICU as well as intensivist perception regarding the use of NMB. As secondary outcomes, we decided to evaluate factors associated with frequent use of NMB during EI and to describe common practices during EI and airway management.

## METHODS

The study protocol was approved by the Research Ethics Committee of *Faculdade de Medicina da Universidade de São Paulo* (number 14637519.2.0000.0065). Participation in the survey was strictly voluntary.

The questionnaire was designed using an informal Delphi process among all authors. One of the authors designed the survey and was responsible for facilitating communication between authors. An initial draft was distributed to all study authors, and responses were compiled into a new draft. Subsequently, the responses were once again reviewed by all authors, and this process was repeated until a consensus was reached with the final questionnaire.

This was an adult ICU physician-centered survey and consisted of 28 questions (Table 1S - Supplementary material). The survey aims were to describe demographic characteristics of the participants and their usual practice with airway devices; to describe the availability of airway resources at the participants’ workplace; to evaluate self-reported use of NMB during EI and perception of the possible benefits associated with the use of NMB; to study which drugs are more commonly prescribed during EI in patients with and without hemodynamic instability, and describe physicians’ strategies in a “do not intubate, do not ventilate” scenarios using clinical vignettes.

Most of the questions in this survey allowed only a single answer. However, participants were able to give multiple answers to questions regarding sedative agents and neuromuscular blockers commonly used during EI so that some percentages could be greater than 100%.

This study was conducted with logistics support from AMIBnet (the Brazilian network of research in ICUs), and the survey was sent to several email lists and those subscribed to the AMIBnet mailing list. Respondents were asked to complete the survey and invited to forward it to a colleague. Reminders were sent after 1 month.

Our primary outcome was to evaluate the use of NMB during EI and physicians’ perceptions regarding possible benefits of the use of NMB during airway management. Secondary outcomes included evaluation of factors associated with the use of NMB during EI, the description of drugs commonly used during EI in patients with and without hemodynamic instability, available airway resources in respondents’ ICUs and the respondents’ demographic data.

### Statistical analysis

Considering the population of 10,000 ICU physicians in Brazil in accordance with a previously reported census from *Associação de Medicina Intensiva Brasileira* (AMIB), we estimated that a sample size of 370 responses would be enough to represent the intended population with a confidence level and a confidence interval of 95% and 5%, respectively.

Continuous data were reported as the mean (standard deviation) and median (25th percentile, 75th percentile) as appropriate. Categorical variables were presented as absolute numbers and percentages.

We used a logistic regression model to assess the association between regular use of NMB in EI and independent variables as follows: the response to the question “How often do you use neuromuscular blockade during EI”? was categorized “as frequent” use and “infrequent” use and adopted as the dependent variable. The Likert scale was used for questionnaire responses; never used, rarely used and regularly used were categorized as “infrequent” use, and frequently and always used were categorized as “frequent use”. We included variables associated with previous experience in critical care, such as board certification in critical care, average number of EIs performed monthly, time since graduation, last medical residency and any previous difficult airway course, as independent variables. The number of EIs was categorized as lower or higher than 3 EIs/month for the analysis. We chose the independent variables to be included in the model based on clinical relevance. We determined the model with the best fit using the Bayesian information criteria and the final model calibration was determined by the Hosmer-Lemeshow test using deciles and the *Gruppo Italiano per la Valutazione Degli Interventi in Terapia Intensiva* (GiViTi) calibration belt. We respected the minimum of one variable to 10 outcomes proportion in model building.

A two-sided p value < 0.05 was considered statistically significant. Odds ratios (OR) and 95% confidence intervals were calculated for associated measurements. The commercial statistical software package used was STATA version 15.1.

## RESULTS

### Sample characteristics

**Figure 1:**
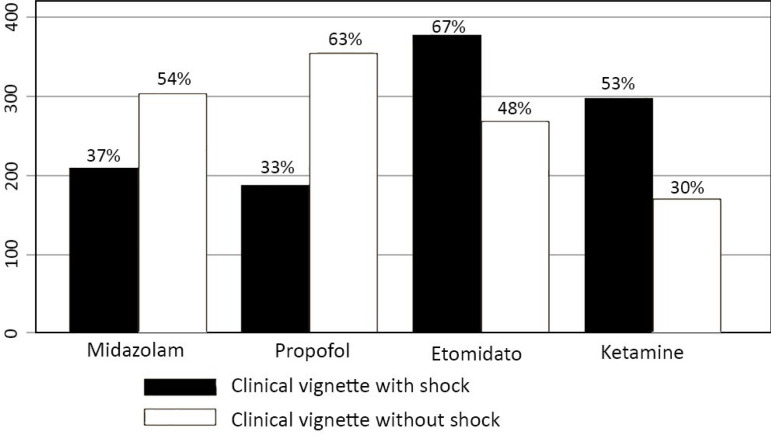
Percentages of responses indicating sedative use during endotracheal intubation in a clinical scenario with (black bars) and without hemodynamic instability (white bars)

Six hundred and twenty-four physicians responded to the survey. However, after removal of incomplete and duplicated responses, 565 remained for final analysis. All regions from Brazil were properly represented in this survey (Figure 1S - Supplementary material). The surveyed workforce was predominantly male (65%), with a mean age of 38 ± 8.4 years. Sixty-eight percent previously attended a difficult airway course, and 403 (71%) reported feeling confident or very confident in managing a difficult airway situation. Physician demographic data are described in table 1. Most responders worked predominantly in a private ICU (52.4%) with a medical-surgical profile (62.7%). Rescue devices such as supraglottic devices were not available in all ICUs (Table 2).

**Table 1:** Characteristic of survey responders

Variables	
Male sex	368 (65)
Age (years)	38 (± 8.4)
Time since graduation (years)	12 (± 8)
Medical residency	
Critical care	235 (41.5)
Internal medicine	169 (30)
Surgery	32 (5.6)
Anesthesiology	9 (1.6)
Board certification in critical care	330 (58.5)
Orotracheal intubation performed monthly	
< 3	149 (26.3)
≥ 3	416 (73.6)
Previous difficult airway course	384 (68)
Confidence in managing a difficult airway	
Confident or very confident	403 (71.3)
Indifferent	57 (10)
Little or very little confidence	105 (18.6)

Results expressed as n (%) or means (± standard deviations).

**Table 2:** Intensive care unit characteristics

Variables	
ICU profile	
Medical	140 (24.7)
Surgical	51 (9)
Trauma	20 (3.5)
Mixed	354 (62.6)
Private ICU	296 (52.3)
Difficult airway kit available	403 (71)
Available devices	
Laryngeal mask	463 (82)
Gum elastic bougie	367 (65)
Video laryngoscopy	150 (26.5)
Bronchoscopy	40 (7)

ICU - intensive care unit. Results expressed as n (%) or means (± standard deviations).

### Use of neuromuscular blockade and factors associated with its use

Only 40.7% of the intensivists reported the use of NMB during all or more than 75% of EI procedures. Thirty-seven percent of the physicians reported using NMB in less than 25% of the procedures. Ninety-one percent of responders described that the use of NMB facilitates the visualization of the vocal cords during EI. In contrast, 22.3% reported that its use could increase the procedural risk.

In the multivariate analysis, the number of EIs performed monthly and physician specialization in anesthesiology were directly associated with frequent use of NMB during EI. In contrast, time (in years) since graduation was inversely associated with frequent use of NMB (Table 3).

**Table 3:** Variables associated with a reported frequent use of neuromuscular blockers during intubation in critically ill patients

Variables	Odds ratio	95%CI	p value
Intubations/month			
< 3	REF		
≥ 3	1.72	1.13 - 2.61	0.011
Years since graduation			
5 - 10	REF		
< 5	1.16	0.62 - 2.16	0.636
10 - 20	0.50	0.33 - 0.78	0.002
20 - 30	0.36	0.19 - 0.66	0.001
≥ 30	0.28	0.11 - 0.68	0.005
Last medical residency			
None	REF		
Anesthesiology	12.76	1.45 -112.49	0.022
Critical care	1.24	0.68 - 2.25	0.488
Internal medicine	1.29	0.73 - 2.29	0.384
General surgery	1.63	0.68 - 3.93	0.275
Other	0.80	0.31 - 2.06	0.647
Board certification	1.08	0.68 - 1.73	0.736
Difficult airway course	1.24	0.84 - 1.82	0.283

95%CI - 95% confidence interval; REF: reference. Calibration - Hosmer-Lemeshow goodness of fit: p value = 0.4893; calibration belt (*Gruppo Italiano per la Valutazione Degli Interventi in Terapia Intensiva*): p-value = 0.444. Discrimination - *area under the Receiver Operating Characteristic* = 0.6547

### Attitude towards a “do not intubate, do not ventilate” scenario

In a “do not intubate, do not ventilate” scenario, 82% of the responders reported the use of a supraglottic device as a rescue strategy. Nine percent and 7.8% reported a new attempt of direct laryngoscopy and urgent cricothyroidotomy as rescue therapy, respectively.

### Attitudes on the use of drugs during airway management

Etomidate and ketamine were the most commonly prescribed drugs in the clinical scenario of shock with the need for vasoactive drugs, while propofol and midazolam were more frequently used in the clinical scenario without hypotension (Figure 1). Sixty-seven percent and 72% of responders reported fentanyl use in the clinical scenario with and without shock, respectively. Succinylcholine and Rocuronium were the most commonly reported NMBs for EI (82.1 and 46.1% of the intensivists, respectively).

## DISCUSSION

The main findings of this survey are summarized as follows: Brazilian critical care physicians report a low use of NMB during EI in the ICU; not all ICU physicians reported the use of supraglottic airway devices as a first-line rescue strategy in a “do not intubate, do not ventilate” scenario; and fentanyl is widely used during EI, and the choice of sedative drugs may vary in accordance with patient hemodynamic status. Taken as a whole, these data suggest there is substantial room for improvement in intubation practices and suggest clinical equipoise for clinical trials assessing the use of neuromuscular blockers and fentanyl in ICU intubations.

The finding that most intensivists do not use NMB regularly during EI in the ICU is not in accordance with published guidelines recommending the routine use of NMB.^(5,6)^ Although a recently published meta-analysis of 34 trials evaluating the impact of NMB in tracheal intubation found that avoidance of NMB was associated with increased risk of patient discomfort, airway injury and difficult laryngoscopy,^(8)^ we must highlight that current evidence is focused mainly on the operating room. Data on emergency departments and critical care units are still sparse. Two observational cohort studies found a reduction in procedure-related complications with the use of NMB during EI in the ICU.^(11,15)^ Similarly, in a propensity matched analysis, the use of NMB improved first attempt success from 69.5% to 80.9% in a cohort of critically ill patients.^(10)^ However, to date, no randomized clinical trial has evaluated the use of NMB in the critical care setting, which could contribute to clinicians’ attitudes towards its use, especially non-anesthetists and physicians who do not intubate frequently.

Our results differ from those of previously published studies. In an Australian and New Zealand survey regarding airway management, most physicians reported the use of rapid sequence intubation in association with NMB in all or in the majority of EI in the ICU.^(16)^ Similarly, in a multicenter national cohort in Scotland, NMB was withheld in only 8% of the EI.^(13)^ In this survey, although 91% of responders reported that the use of NMB facilitates EI in the ICU, 22% also reported that its use could increase procedure-associated risk, suggesting a fear of a “do not intubate, do not ventilate” scenario and its consequences. A possible explanation for this difference is the fact that intensive care physician training is associated with anesthesia departments in several countries and, therefore, practices from the operating room may be more easily transposed to the ICU. In Brazil, most intensivists are not anesthetists by training, and in our survey, only a minimum proportion of responders were anesthetists. Despite the small number of responses, anesthesia training was directly associated with frequent use of NMB in the multivariate analysis. Additionally, a higher number of EIs performed monthly was directly associated with the use of NMB, suggesting that the more experienced the physician, the higher the probability of using NMB.

In regard to sedative drugs and analgesics used during EI, we found that etomidate and ketamine were more commonly used in the clinical situation of hypotension and shock. On the other hand, propofol and midazolam were more commonly prescribed in the situation of clinical stability. This is endorsed by previous recommendations in which the choice of induction drug should be dictated by patient hemodynamic status.^(5)^ Moreover, we found a higher report of succinylcholine use than rocuronium use in our survey. Although some recommendations suggest that rocuronium should be preferred over succinylcholine in EI, a randomized controlled trial found both drugs to be equivalent in the ICU.^(17)^

To our knowledge, this is the first large survey regarding airway management, the use of sedatives and NMB in Brazil. Furthermore, these results may help the design of future research regarding airway management in Brazil along with the standardization of practices to allow a safer procedure. However, our study has several limitations. First, a selection bias is always a possibility in a survey, and we did not assess the nonresponse rate. It is possible that nonresponders had a different prescribing practice. We reached a highly qualified sample with more than half of the responders having board certification, which may not represent the intended population. However, we reached a response rate higher than initially expected, which could minimize these risks. Second, since patient medical records were not assessed, a certain degree of recall bias may have influenced physicians’ responses. Third, most responses were from the Southeast region of Brazil, which may limit the generalizability of the data. However, all regions were properly represented in accordance with the national ICU physician’s registry.

## CONCLUSION

The reported use of neuromuscular blockers is low among intensivists, and almost a quart of physicians raised the concern of increased risk with the use of neuromuscular blockers. Fentanyl use was not determined by the patient’s hemodynamic status, and sedative drugs were chosen according to the patient’s hemodynamic status. The availability of different airway devices, as well as physicians’ knowledge and confidence regarding difficult airway management, was heterogeneous.
