# Supplementary material for: Neuromuscular blockade and airway management during endotracheal intubation in Brazilian intensive care units: a national survey
Source: Rev Bras Ter Intensiva. 2020 Jul-Sep;32(3):433–8. doi: 10.5935/0103-507X.20200073 (PMC7595723; doi:10.5935/0103-507X.20200073)
Supplement: Supplementary file 1 [file rbti-32-03-0433-suppl01.pdf]

Neuromuscular blockade and airway management during endotracheal intubation in Brazilian intensive care units: a national survey

Bloqueio neuromuscular e manuseio das vias aéreas na intubação endotraqueal em unidades de terapia intensiva brasileiras: um levantamento nacional

Pedro Vitale Mendes[1,2], Bruno Adler Maccagnan Pinheiro Besen[1,3], Fabio Holanda Lacerda[1], João Gabriel Rosa Ramos[4], Leandro Utino Taniguchi[1,5]

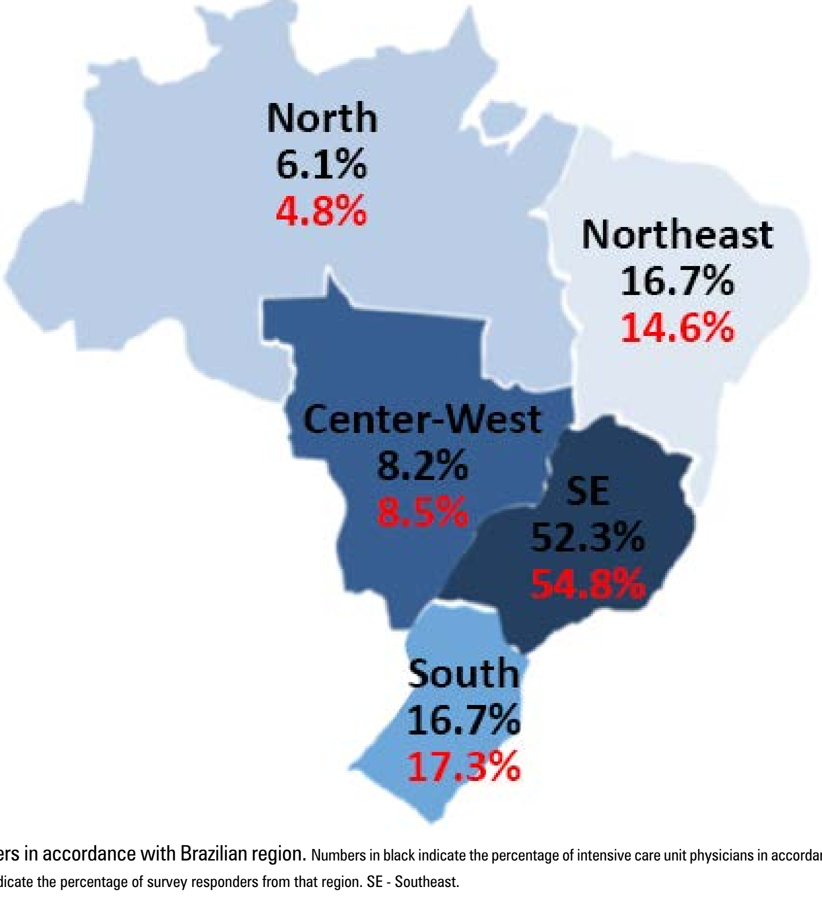

Figure 1S - Distribution of responders in accordance with Brazilian region. Numbers in black indicate the percentage of intensive care unit physicians in accordance with the latest *Associação de Medicina Intensiva Brasileira* census. Numbers in red indicate the percentage of survey responders from that region. SE - Southeast.

Table 1S - Summary of survey questions

1. Name
2. Birth date
3. Sex
4. Graduation year (medical school)
5. Have you concluded medical residency?
6. Specify which medical residency
7. Do you have critical care board certification?
8. How long have you worked in the intensive care unit (including specialization)?
9. How many hours per week do you usually work in an intensive care unit?
10. In which Brazilian state do you work?
11. How many endotracheal intubations (on average) do you perform monthly?
12. Have you used any supraglottic devices in the past 3 months?
13. How many emergency cricothyroidotomies have you performed in your medical career?
14. Have you ever participated in a formal airway course/training?
15. How confident do you feel in managing a difficult airway?
16. Which sedative drugs would you use for endotracheal intubation in the following clinical scenario patient with urinary tract infection and septic shock who needs 0.3mcg/kg/min of noradrenaline?
17. Which sedative drugs would you use for endotracheal intubation in the following clinical scenario: patient with pneumonia and acute respiratory distress, without hemodynamic instability?
18. How often do you use neuromuscular blockade for endotracheal intubation in the intensive care unit?
Never // Rarely // Regularly // Frequently // Always
19. In your opinion, the use of neuromuscular blockade in endotracheal intubation may increase or reduce the procedure-associated risk?
20. In your opinion, the use of neuromuscular blockade in endotracheal intubation may facilitate difficult vocal chord visualization?
21. In your opinion, the use of neuromuscular blockade in endotracheal intubation may facilitate or difficult bag-valve-mask ventilation?
22. Which neuromuscular blocker do you more commonly use in endotracheal intubation?
23. Which rescue strategy would you adopt in the following case:
Sixty-year-old patient in acute respiratory distress. After an unsuccessful intubation attempt with optimal positioning and sedation strategy you initiate bag-valve mask ventilation, but oxygen saturation keeps progressively falling.
    - Keep bag-valve-mask ventilation.
    - New EI attempt with direct laryngoscopy.
    - Use a supraglottic device.
    - Emergency cricothyroidotomy.
24. Do you work in a private or public intensive care unit most of the time?
25. Do you usually help less experienced physicians during endotracheal intubation?
26. Which devices are available for use in your intensive care unit?
    - Gum elastic bougie.
    - Flexible laryngoscope blade.
    - Video laryngoscopy.
    - Laryngeal mask or similar device.
    - Fast track laryngeal mask.
    - Laryngeal tube.
    - Optic devices.
    - Bronchoscopy (available for use by the intensivist in the intensive care unit).
27. Does your intensive care unit have an easy access to a difficult airway kit?
28. Please, select the type of the intensive care unit in which you work most of the time.
    - Medical intensive care unit.
    - Surgical intensive care unit.
    - Trauma intensive care unit.
    - Mixed intensive care unit.
